# The Impact of Exercise Training in a Hypobaric/Normobaric Hypoxic Environment on Cardiometabolic Health in Adults with Overweight or Obesity: A Systematic Review and Meta-Analysis

**DOI:** 10.3390/life15040566

**Published:** 2025-03-31

**Authors:** Peng Liu, Hao Chen, Yidi Deng, Xin Jiang

**Affiliations:** 1 College of Physical Education, Dalian University, Dalian 116622, China; liupeng@s.dlu.edu.cn (P.L.); dengyidi@s.dlu.edu.cn (Y.D.); 2 Physical Education Department, Dalian University of Finance and Economics, Dalian 116622, China; chenhao@dlufe.edu.cn; 3 Graduate School, Beijing Sport University, Beijing 100084, China

**Keywords:** hypoxic training, normoxic training, obesity, cardiometabolic health, meta-analysis

## Abstract

This systematic review and meta-analysis aims to comprehensively evaluate the effects of hypoxic training (HT) versus normoxic training (NT) on cardiometabolic health parameters in overweight or obese adults. Searches were performed in PubMed, Web of Science, Embase, Scopus, and the Cochrane Library. A meta-analysis was performed using Stata 18 and RevMan 5.4 software. Seventeen randomized controlled studies involving 517 participants were included. HT significantly improved cardiorespiratory fitness (CRF) and reduced systolic blood pressure (SBP) and diastolic blood pressure (DBP). Compared with NT, HT demonstrated a significant difference in CRF, but no significant differences were observed in SBP and DBP. The subgroup analysis of CRF revealed that HT significantly outperformed NT in six aspects: participants aged < 45 years (Hedges’ g = 0.50), an intervention duration of 8 weeks (Hedges’ g = 0.43), three sessions per week (Hedges’ g = 0.40), each session lasting < 45 min (Hedges’ g = 0.23), FiO_2_ levels > 15% (Hedges’ g = 0.69), and high-load-intensity exercise (Hedges’ g = 0.57). HT demonstrated favorable effects in improving cardiometabolic health among overweight or obese adults. Compared with NT, this advantage was primarily reflected in CRF while the impacts on SBP and DBP were similar.

## 1. Introduction

Obesity is internationally recognized as a complex chronic disease [1]. Studies indicate that the global prevalence of obesity among adults nearly doubled between 1999 and 2021, and projections suggest that, by 2050, over 3.8 billion adults aged 25 and older worldwide will be affected by obesity [2]. Obesity is associated with a decline in cardiorespiratory fitness and an excessive accumulation of visceral fat, both of which are independent risk factors for cardiovascular disease (CVD) [3]. CRF is a critical marker of cardiovascular risk and mortality [4]. VO_2_max is one of the core indicators of CRF [5]. It not only reflects the ability of the respiratory and circulatory systems to supply oxygen to muscles during prolonged exercise [6] but is also widely recognized as a robust independent predictor of cardiovascular disease risk and mortality [7,8]. Researchers can measure the VO_2_max through a cardiopulmonary exercise test (CPET) [9]. The CPET has witnessed a growing prevalence in clinical research and sports performance, and it is also considered as the gold standard for evaluating CRF and exercise capacity [10]. VO_2_max exhibits an inverse correlation with the incidence of cardiovascular disease and all-cause mortality [11]. Obese individuals frequently experience hypertension, resulting in elevated systolic blood pressure (SBP) and diastolic blood pressure (DBP). SBP is an independent risk factor for cardiovascular disease [12].

Insufficient physical activity and sedentary behavior are strongly associated with increased risks of all-cause mortality, cardiovascular mortality, and cancer mortality [13]. Exercise is a potent non-pharmacological intervention for the treatment and prevention of a variety of chronic diseases [14], as well as an effective strategy for the prevention and management of obesity [15]. Regular exercise not only enhances cardiovascular health [14,16,17] and cardiometabolic function but also induces anabolic processes (e.g., increased skeletal muscle anabolism) and metabolic adaptations, thereby reducing mortality rates and improving quality of life [18,19,20]. Exercise can also increase the secretion of endorphins, regulate emotions, and enhance the treatment compliance of patients [21]. Numerous studies have demonstrated that exercise can reduce body mass index (BMI) in overweight and obese adults, decrease cardiovascular disease risk, and improve cardiometabolic health [22,23]. For example, aerobic training of moderate intensity improved an individual’s cardiopulmonary health [22]. The intensity and duration ranges were 60–70% of VO_2_max and 60–90 min, respectively [24]. HIIT features brief high-intensity exercise bouts alternating with low-intensity passive or active recovery periods [25]. HIIT can generate a potent stimulus to increase the gene expression involved in mitochondrial biogenesis and the regulation of oxidative enzymes in skeletal muscle [26]. HIIT has been shown to improve physiological and psychological adaptations related to cardiometabolic health in overweight and obese adults [27,28]. Additionally, resistance training combined with aerobic or endurance training can reduce fat mass and promote cardiometabolic benefits in obese individuals [29,30,31].

Hypoxia is defined as a reduction in arterial oxygen saturation, resulting in decreased oxygen supply to tissues [32]. In recent years, hypoxic exposure, also known as hypoxia conditioning, has gained increasing attention as a novel therapeutic strategy for enhancing human health [33]. Studies have demonstrated that hypoxic environments provide unique benefits, including weight loss, increased energy expenditure, enhanced fat oxidation, and improved cardiovascular function [27,34,35]. Hypoxic conditions lead to an increase in the diameter of arterioles, induce peripheral vasodilation, and result in a reduction in arterial blood pressure [21]. As an effective non-pharmacological intervention, HT does not induce side effects in obese individuals [36]. Instead, it can promote significant weight loss through negative energy balance while enhancing exercise adherence [37]. HT can confer various health benefits for overweight or obese individuals, including reduced fat mass, improved lipid profiles, regulated blood glucose levels, and enhanced insulin sensitivity [24]. The underlying mechanism can be attributed to the fact that HT has the potential to augment mitochondrial quantity, capillary density [38], the activity of glycolytic enzymes, and the expression level of glucose transporter GLUT-4 [21]. Additionally, HT has been associated with significant improvements in cognitive function in both healthy individuals and the elderly [39,40]. HT has also been shown to effectively enhance VO_2_max in athletes [41,42]. Some studies have suggested that HT does not lead to significant improvements in cardiorespiratory function in obese populations [5]. However, other studies have demonstrated that HT can significantly enhance CRF [43]. Given that CRF is a critical marker of cardiovascular risk and mortality, it is essential to synthesize existing evidence through systematic reviews and meta-analyses to elucidate the effects of HT on cardiometabolic health in overweight or obese populations.

To date, only one systematic review and meta-analysis has investigated the effects of normobaric hypoxia training on cardiometabolic health in overweight or obese populations; however, it did not evaluate the benefits of CRF indicators [23]. Building on the previous research, this study aims to evaluate the effects of hypobaric/normobaric hypoxia training on cardiometabolic health in overweight or obese adults through a meta-analysis. Meanwhile, it intends to elucidate its advantages over normoxic training and further provide more precise exercise prescriptions for this population.

## 2. Materials and Methods

### 2.1. Design

This study was conducted in accordance with the PRISMA guidelines [44]. This study was registered in PROSPERO under the registration number CRD420251003429.

### 2.2. Literature Search

A comprehensive systematic search was conducted across five databases—PubMed, Web of Science, Embase, Scopus, and the Cochrane Library—for studies published from the inception of each database until 22 February 2025. Relevant search terms were combined using Boolean logic operators (AND, OR) to refine the search strategy. The specific search strategies and results are provided in Table S1. Additionally, the reference lists of the included studies and relevant meta-analyses were manually screened to identify potentially eligible studies that were omitted from the initial search.

### 2.3. Inclusion and Exclusion Criteria

The eligible studies were selected independently by two authors (HC and PL). Disagreements were resolved by a third author (XJ). The inclusion criteria were based on the PICOS principles: (1) The participants included overweight or obese adults aged ≥ 18 years with no physical constraints or health circumstances that would impede the evaluation and the exercise-related intervention. (2) The experimental group underwent training under hypobaric or normobaric hypoxia conditions, whereas the control group trained under normoxic conditions. (3) The hypoxic environment was defined as an FiO_2_ ≤ 17.4% or an altitude ≥ 1500 m. (4) Only randomized controlled trials (RCTs) were included. (5) The outcome measures comprised CRF (including VO_2_max/VO_2_peak), SBP, DBP, with at least one of these outcomes reported in the selected study.

The exclusion criteria were as follows: (1) studies not involving overweight or obese adults; (2) reviews, systematic reviews, and meta-analyses; (3) full-text articles unavailable; (4) full-text articles lacking the specified outcome measures; (5) studies irrelevant to the research topic; (6) studies from which outcome measures could not be extracted; and (7) non-English language studies. In the conduct of this review, only English-language studies were included. Non-English studies, even if they held valuable information, were excluded on account of the challenges in translation and ensuring correct interpretation. Failure to address these challenges properly could lead to biases in the research outcomes.

### 2.4. Literature Screening and Data Extraction

The predefined search strategy was applied to retrieve studies from the databases, and the resulting literature was imported into EndNote X9 software for duplicate removal. The titles of the retrieved studies were screened to exclude the irrelevant literature. Subsequently, the abstracts or full texts were evaluated against the inclusion and exclusion criteria to exclude studies that did not meet the eligibility criteria.

The primary data extracted from the included studies comprised the following: first author, publication year, experimental groups, age and gender of participants, sample size, inclusion criteria (BMI), oxygen concentration/altitude, intervention protocol, load intensity, training duration, training frequency, and outcome measures. In studies in which the standard error (SEM) was reported, the standard deviation (SD) was calculated using the equation SD = SEM × $\sqrt{N}$, where SD represents the standard deviation, SEM denotes the standard error of the mean, and *N* refers to the sample size. Two authors (HC and PL) extracted the data using a specific sheet, and any disagreements were resolved through discussion with a third author (XJ).

### 2.5. Risk-of-Bias Assessment for Included Studies

The Cochrane Collaboration’s tool for assessing the risk of bias in randomized controlled trials (RCTs) was used to evaluate the following domains: (1) random sequence generation; (2) allocation concealment; (3) blinding of participants and personnel; (4) blinding of outcome assessment; (5) incomplete outcome data; (6) selective reporting; and (7) other potential sources of bias. The assessment of the risk of bias was carried out independently by two authors (PL and YD), and any discrepancies were resolved through discussion.

### 2.6. Certainty of Evidence

The Grading of Recommendations Assessment, Development, and Evaluation (GRADE) method was employed to assess the quality of evidence [45]. GRADE assesses the certainty of evidence falling into the categories of very low, low, moderate, or high.

### 2.7. Statistical Analysis

Meta-analysis, sensitivity analysis, subgroup analysis, and publication bias assessment were conducted on the outcome measures of the included studies using Stata 18 and RevMan 5.4 software. The outcome measures in the included studies were continuous variables. When the units of the outcome measures were consistent, the mean difference (MD) was used as the effect measure, assuming unequal variances between groups. When the units of the outcome measures were inconsistent, Hedges’ g was used as the effect measure, with the exact calculation method applied to compute the bias correction factor and the Hedges and Olkin correction used to calculate the standard error of the effect size. The magnitude of the effect size was interpreted as follows: negligible (<0.2), small (0.2–0.5), medium (0.5–0.8), and large (>0.8). The heterogeneity of the outcome measures was evaluated using the *I*^2^ statistic and *p*-value. If *I*^2^ < 50% and *p* > 0.1, indicating low heterogeneity among the studies, a fixed-effects model with inverse variance was applied. Otherwise, a random-effects model using the DerSimonian–Laird method was employed. A *p*-value < 0.05 was considered statistically significant. If substantial heterogeneity was observed, the stability of the results was assessed using the Leave-One-Out method. Publication bias was evaluated using funnel plots or Egger’s test. If the funnel plot exhibits asymmetry or the Egger’s test yields a *p*-value < 0.05, it is considered indicative of significant publication bias. Conversely, the absence of these findings suggests no significant publication bias.

## 3. Results

### 3.1. Subsection

**Figure 1:**
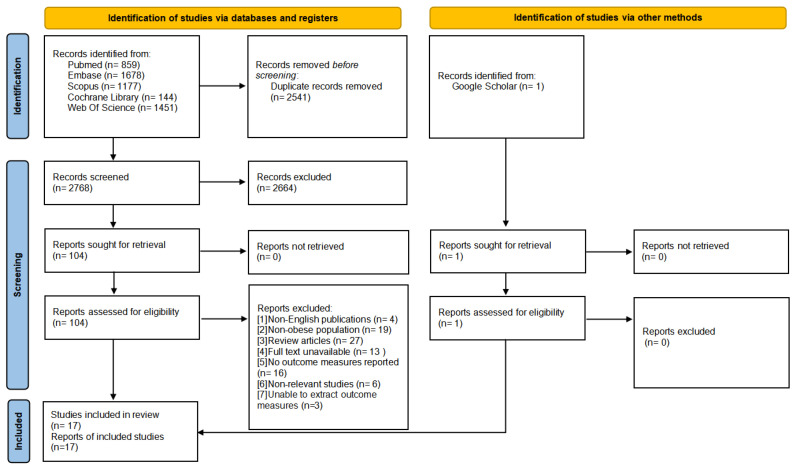
PRISMA flowchart of study selection.

A total of 5309 articles were initially retrieved from the databases. After excluding 2541 duplicate studies and 2664 irrelevant studies, a comprehensive evaluation was conducted on the remaining 104 articles. Ultimately, 17 studies met the inclusion criteria and were included in the final analysis. The flowchart of the literature screening process is presented in Figure 1.

### 3.2. Study Characteristics

The basic characteristics of the included studies are summarized in Table S2. A total of 517 overweight or obese adults were included in the analysis HT: 267, NT: 250. Two studies involved male participants; six studies involved female participants; eight studies included both male and female participants; and one study did not report the gender of the participants. The hypoxic conditions in the included studies ranged from 17.2% to 12% FiO_2_. The interventions in the studies included walking (*n* = 2), aerobic exercise (*n* = 7), high-intensity interval training or high-intensity full sprint (*n* = 4), combined aerobic and resistance training (*n* = 2), combined training (*n* = 1), and Pilates (*n* = 1). Twelve studies reported CRF and fourteen studies reported SBP and DBP.

### 3.3. Risk-of-Bias Assessment Results

**Figure 2:**
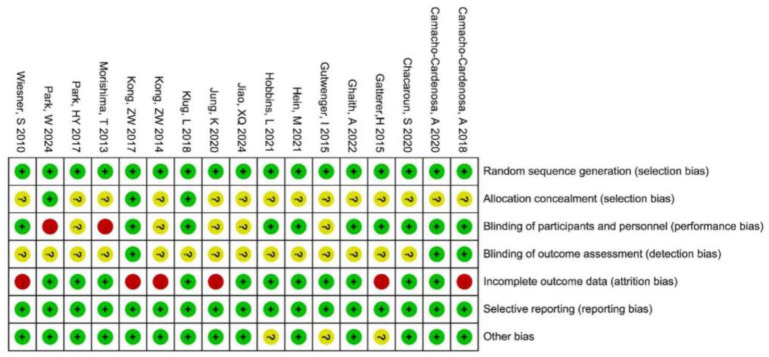
The risk assessment of bias [5,27,43,46,47,48,49,50,51,52,53,54,55,56,57,58,59].

The risk-of-bias assessment for the included studies is presented in Figure 2. Three studies explicitly described allocation concealment. Regarding blinding, seven studies employed a single-blind design, and three studies utilized a double-blind design. Six studies reported issues with participant dropout during the intervention period. Two studies had small sample sizes, with fewer than 10 participants in the intervention and control groups. One long-term intervention study exhibited discrepancies between the actual tested sample size and the reported sample size.

### 3.4. Certainty of Evidence

The overall certainty of evidence underwent assessment with the application of the GRADE tool, and the findings are presented in Table S3. H-post represents the measured value after HT; H-pre represents the measured value before HT; and N-post indicates the measured value after NT. The GRADE method shows that the certainty level of DBP (H-post, H-pre) was moderate, and the certainty level of CRF and SBP was low. The level of certainty for DBP (H-post, N-post) was very low.

### 3.5. Meta-Analysis

#### 3.5.1. CRF

**Figure 3:**
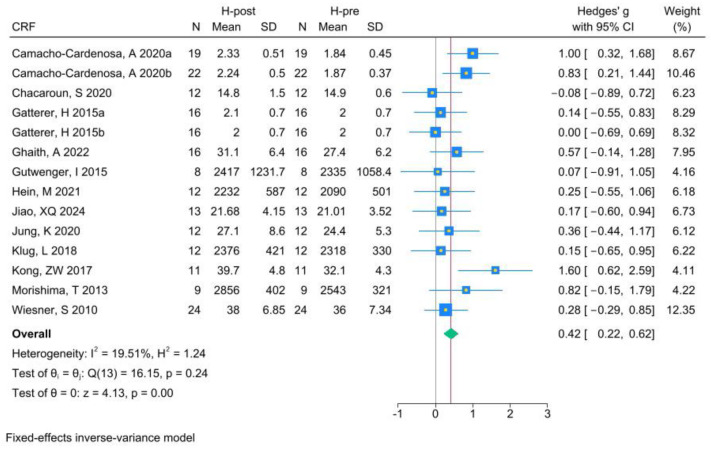
Forest plot of CRF (H-post, H-pre) meta-analysis [5,43,46,47,48,49,50,51,52,53,54,55].

**Figure 4:**
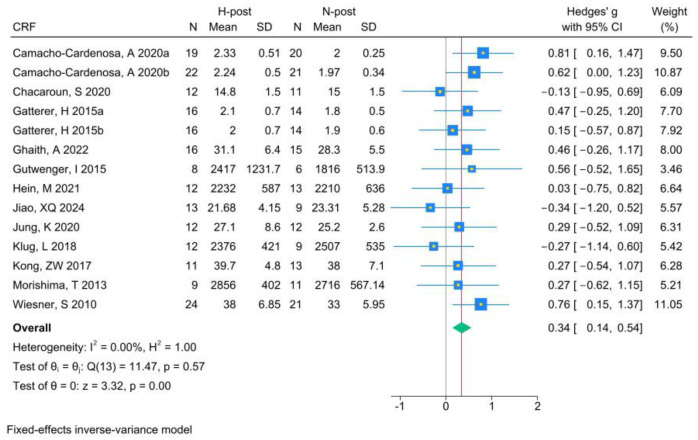
Forest plot of CRF (H-post, N-post) meta-analysis [5,43,46,47,48,49,50,51,52,53,54,55].

The analysis of CRF included 14 RCTs. Analysis of the pre- and post-HT data (see Figure 3 for individual studies) demonstrated that HT significantly improved CRF (Hedges’ g 0.42, 95% CI 0.22 to 0.62; *p* = 0.00, *I*^2^ = 19.51%). Compared with NT (see Figure 4 for individual studies), HT was significantly superior (Hedges’ g 0.34, 95% CI 0.14 to 0.54; *p* = 0.00, *I*^2^ = 0.00%).

#### 3.5.2. SBP

**Figure 5:**
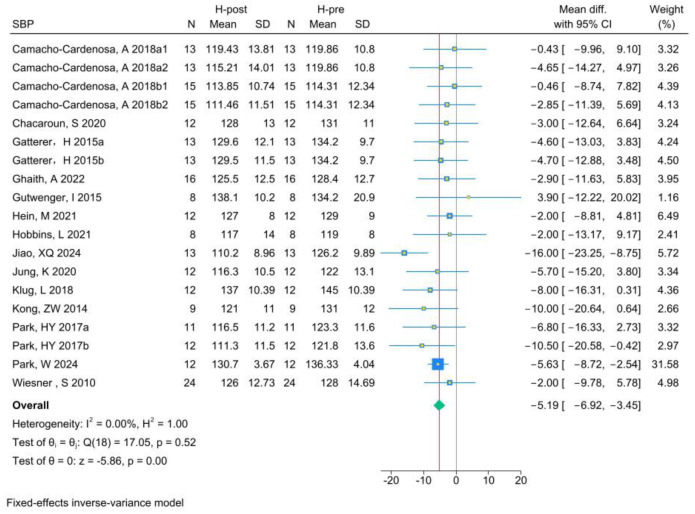
Forest plot of SBP (H-post, H-pre) meta-analysis [5,27,46,49,50,51,52,53,54,55,56,57,58,59].

**Figure 6:**
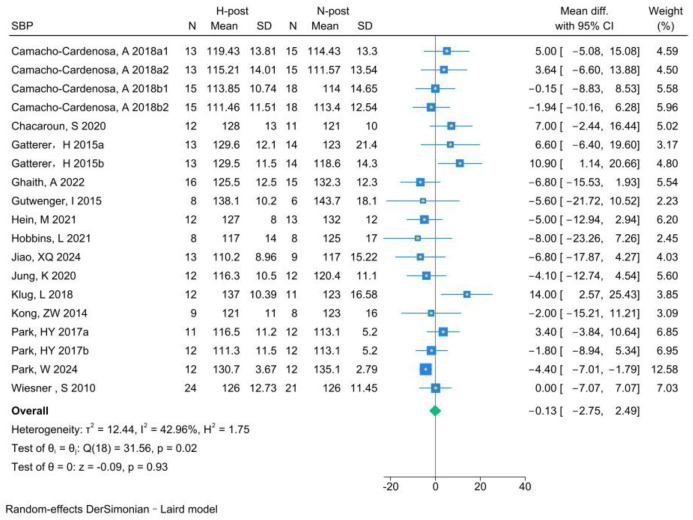
Forest plot of SBP (H-post, N-post) meta-analysis [5,27,46,49,50,51,52,53,54,55,56,57,58,59].

The analysis of SBP included 19 RCTs. Analysis of the pre- and post-HT data (individual studies are presented in Figure 5) demonstrated that HT significantly improved SBP (MD −5.19, 95% CI −6.92 to −3.45; *p* = 0.000, *I*^2^ = 0.00%). Compared with NT (see Figure 6 for individual studies), there was no significant difference between HT and NT (MD −0.13, 95% CI −2.75 to 2.49; *p* = 0.93, *I*^2^ = 42.96%).

#### 3.5.3. DBP

**Figure 7:**
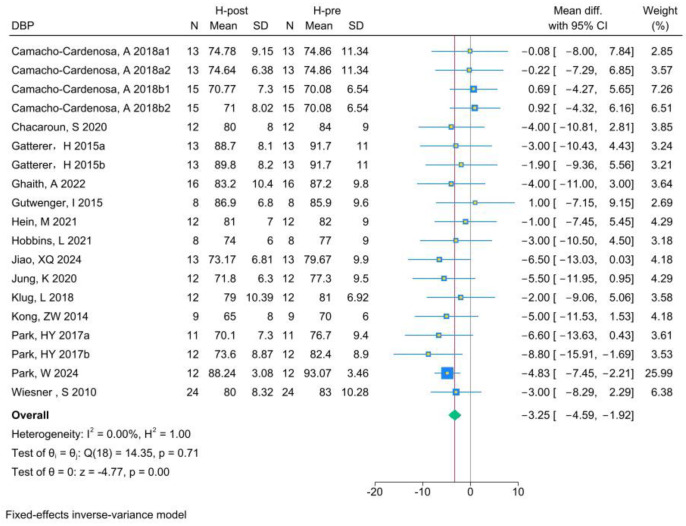
Forest plot of DBP (H-post, H-pre) meta-analysis [5,27,46,49,50,51,52,53,54,55,56,57,58,59].

**Figure 8:**
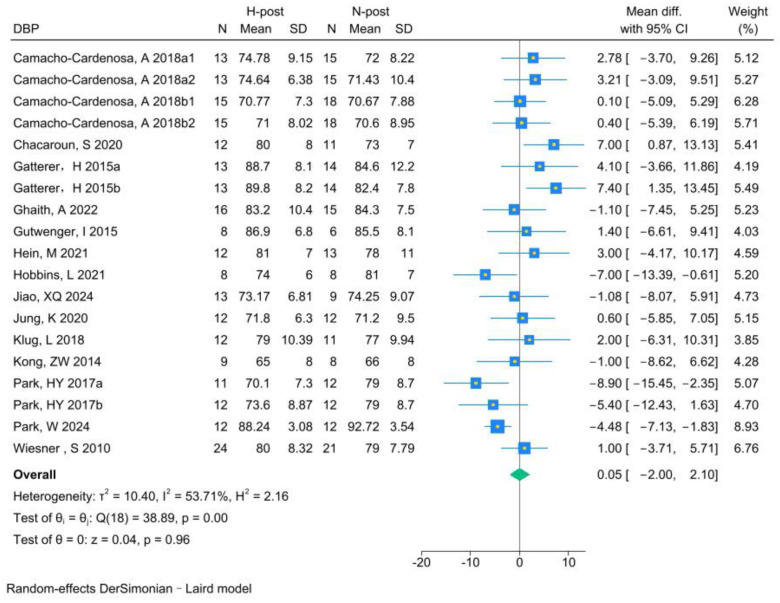
Forest plot of DBP (H-post, N-post) meta-analysis [5,27,46,49,50,51,52,53,54,55,56,57,58,59].

The analysis of DBP included 19 RCTs. Analysis of the pre- and post-HT data (see Figure 7 for individual studies) demonstrated that HT significantly improved DBP (MD −3.25, 95% CI −4.59 to −1.92; *p* = 0.00, *I*^2^ = 0.00%). Compared with NT (see Figure 8 for individual studies), there was no significant difference between HT and NT (MD 0.05, 95% CI −2.00 to 2.10; *p* = 0.96, *I*^2^ = 53.71%).

### 3.6. Sensitivity Analysis

To assess the robustness of the meta-analysis results, a Leave-One-Out sensitivity analysis was conducted for outcomes with high heterogeneity: DBP (H-post vs. N-post). The results demonstrated that, after sequentially excluding individual studies [56], the heterogeneity significantly decreased (MD 0.50, 95% CI −1.49 to 2.48; *p* = 0.62, *I*^2^ = 40%), and the pooled results remained consistent with the original meta-analysis findings.

### 3.7. Subgroup Analysis

**Figure 9:**
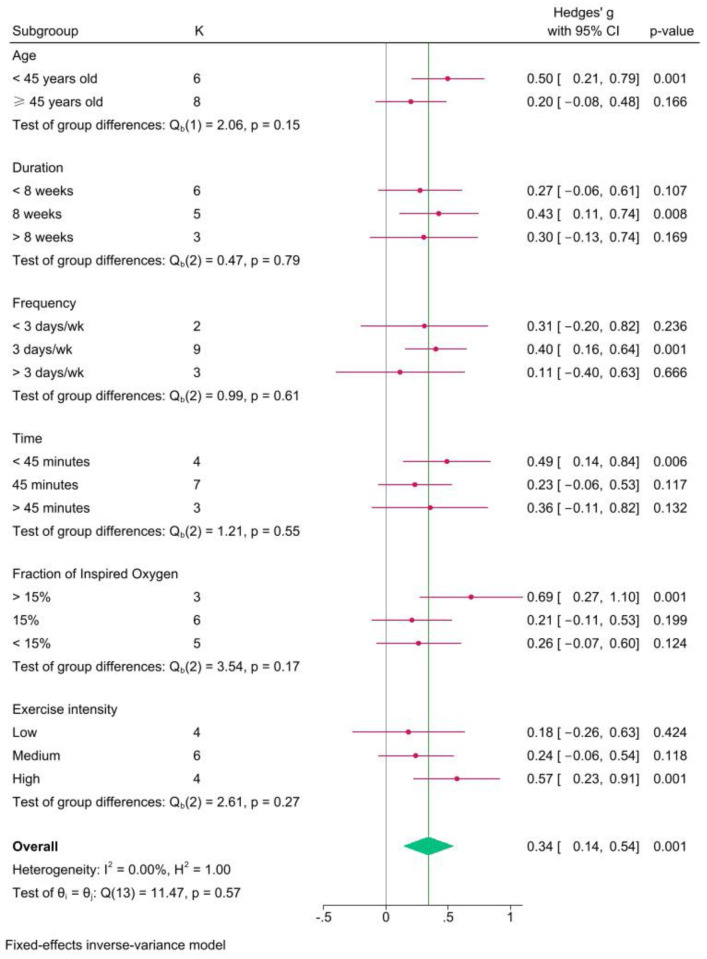
Results of subgroup analysis.

Subgroup analyses were performed for the outcome measure CRF (H-post vs. N-post) based on age, duration, frequency, time, oxygen concentration, and exercise intensity (Figure 9). The subgroup variables included age, duration, frequency, time, FiO_2_, and exercise intensity. The results demonstrated that HT was significantly superior to NT in six aspects: age < 45 years (Hedges’ g = 0.50, *p* = 0.001); intervention duration of 8 weeks (Hedges’ g = 0.43, *p* = 0.008); three sessions per week (Hedges’ g= 0.40, *p* = 0.001); each session lasting< 45 min (Hedges’ g = 0.49, *p* = 0.006); FiO_2_ > 15% (Hedges’ g = 0.69, *p* = 0.001); and high-load-intensity exercise (Hedges’ g= 0.57, *p* = 0.001).

### 3.8. Publication Bias Analysis

**Figure 10:**
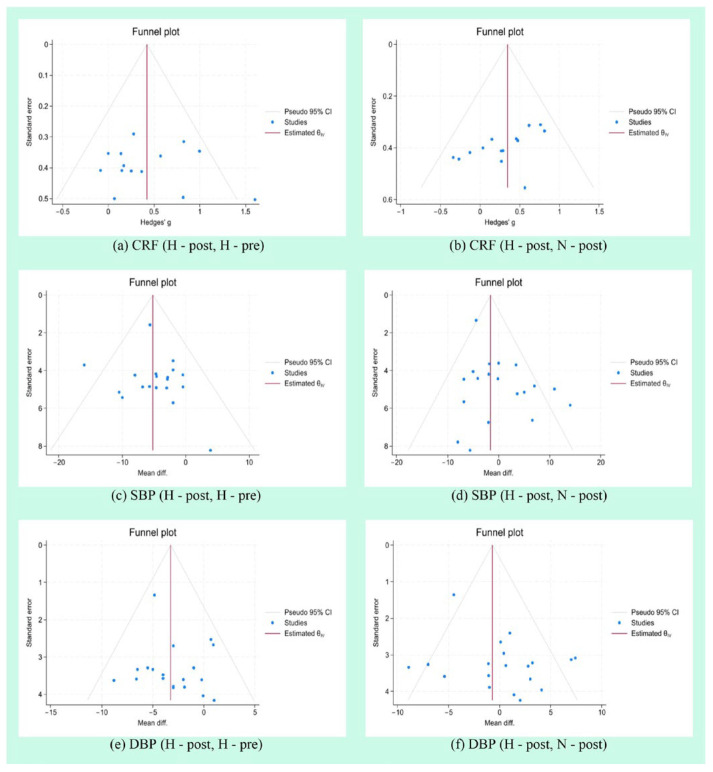
Publication bias analysis result.

The funnel plots for each outcome measure exhibited slight asymmetry (Figure 10). Egger’s regression tests were conducted for each outcome measure. Specifically, the *p*-values were as follows: CRF (H-post, H-pre): *p* = 0.673; CRF (H-post, N-post): *p* = 0.0367; SBP (H-post, H-pre): *p* = 0.5022; SBP (H-post, N-post): *p* = 0.2498; DBP (H-post, H-pre): *p* = 0.422; and DBP (H-post, N-post): *p* =0.2991.

## 4. Discussion

To the best of our knowledge, this is the first systematic review and meta-analysis to comprehensively evaluate the effects of exercise training in hypobaric or normobaric hypoxic environments on cardiometabolic health in overweight or obese adults. This study aimed to assess the impact of hypoxic training on cardiometabolic health in overweight or obese adults through meta-analysis, while also elucidating its advantages compared with normoxic training. Through subgroup analysis, this study provides evidence-based hypoxic exercise prescriptions for overweight or obese adults, thereby contributing to the improvement of cardiometabolic health in this population. The primary finding of this meta-analysis is that HT significantly improved CRF, SBP, and DBP in overweight or obese adults. Compared with NT, HT demonstrated a significant advantage in improving CRF. The subgroup analysis demonstrated that HT was significantly superior to NT in the following six aspects: age < 45 years, an intervention duration of 8 weeks, three sessions per week, each session lasting < 45 min, FiO_2_ > 15%, and high-load-intensity exercise.

The results of this study demonstrate that HT significantly improved CRF in overweight or obese adults (Hedges’ g = 0.42, *p* = 0.00) and demonstrated a significant advantage compared with NT (Hedges’ g = 0.34, *p* = 0.00). Previous studies have primarily focused on the effects of various exercise modalities on cardiometabolic health in overweight or obese adults [30]. Currently, only one meta-analysis on normobaric hypoxic training has investigated its effects on cardiometabolic health in overweight or obese individuals [23]; however, that study did not include any content related to CRF. The results of a network meta-analysis indicated that various hypoxic training paradigms can effectively improve VO_2_max in healthy adults [60], which is consistent with the results of the present study. 

A meta-analysis of the effects of altitude training on the aerobic capacity of athletes demonstrated that HT did not significantly affect VO_2_max but could significantly increase hemoglobin levels in athletes [61]. VO_2_max is one of the most important markers of CRF [5]. The mechanisms underlying hypoxia-induced improvements in VO_2_max are not yet fully understood [62]. A potential mechanism is that the body increases erythropoietin (EPO) production in hypoxic environments, promoting an increase in red blood cell count in the bloodstream [63]. This leads to an increase in hemoglobin levels, enabling the transport of more oxygen to various tissues in the body, thereby enhancing the body’s maximal aerobic metabolic capacity [64]. Compared with hypoxia stimulation alone, exercise combined with a hypoxic environment appears to be more effective in promoting CRF in humans [65], primarily reflected in greater improvements in VO_2_max. The primary reason is that HT enhances the body’s physiological responses and adaptive capacity to hypoxia, thereby improving VO_2_max [62]. This anti-hypoxic physiological response and adaptive capacity can be more effectively mobilized in younger individuals [43], which corroborates the findings of the subgroup analysis in this study, in which, compared with NT, individuals aged < 45 years showed a significant improvement in CRF after HT (Hedges’ g = 0.50). Our analysis found that when FiO_2_ > 15%, HT had a better effect on CRF in overweight or obese individuals (Hedges’ g = 0.69, *p* = 0.001). 

Although current studies have confirmed that moderate- or high-intensity training in hypoxic environments is safe and feasible [48,66], exercising in hypoxic environments also induces greater metabolic responses and increases the level of fatigue in the body [67]. However, studies have demonstrated that the effects of exercise on subjective perceived fatigue are similar in hypoxic and normoxic environments, and there is no gender disparity [68]. When the oxygen concentration is too low, it may also cause harm to the cardiovascular system. Some studies have demonstrated that combining hypoxic stimulation with low- or moderate-intensity training does not lead to significant improvements in CRF [69,70]. When hypoxia is combined with high-intensity training (e.g., >60% HRR, >77% HRmax, >64% VO_2_max), it increases the stimulation of the cardiovascular system, thereby enhancing the body’s adaptive capacity [48]. Simultaneously, under conditions of reduced cellular oxygen supply, lower blood oxygen saturation can trigger the transactivation of hypoxia-inducible factors (HIFs) [71]. High-intensity training can significantly improve brachial artery flow-mediated vasodilation, thereby increasing VO_2_max to a greater extent [72]. Therefore, combining hypoxic stimulation with high-intensity training may lead to greater improvements in CRF. 

Current studies have found that the duration of high-intensity hypoxic training for overweight or obese adults is maintained between 16 and 42 min [28,43,48,59,73], and the intervention period is mostly 12 weeks [28,43,59,73], the frequency of interventions is mostly three times per week [5,28,43,59,73]. This is consistent with the subgroup analysis in this study, in which high-intensity exercise (Hedges’ g = 0.57, *p* = 0.001), each session lasting < 45 min (Hedges’ g = 0.49, *p* = 0.006), and three sessions per week (Hedges’ g = 0.66, *p* = 0.040) significantly improved CRF. Unlike previous findings, the intervention period no longer requires 12 weeks; an 8-week intervention (Hedges’ g = 0.43, *p* = 0.008) can significantly impact CRF in overweight or obese adults. 

A growing body of research indicates that implementing HIIT is beneficial [74,75,76]. HIIT can be divided into high-volume, high-intensity interval training and low-volume, high-intensity interval training according to the cumulative duration of 15 min of high-intensity exercise [77]. Among them, for low-volume, high-intensity interval training, low-intensity recovery exercises are arranged between every two high-intensity exercise sessions. In contrast, for high-volume, high-intensity interval training, the total duration of high-intensity exercises is no more than 15 min, and this time scope does not include the durations of the warm-up phase, the intervals, and the recovery stage [78]. This form of exercise is currently favored by young people, as it does not require a long duration of physical activity [79], achieves significant fat loss results [80,81], and provides substantial benefits for cardiometabolic health [82,83]. Therefore, we recommend that overweight or obese young individuals (aged < 45 years) engage in HIIT under hypoxic conditions (FiO_2_ > 15%) for 8 weeks, with three sessions per week and each session lasting less than 45 min, to enhance CRF and improve cardiometabolic health.

Numerous studies have demonstrated that both individuals with hypertension and those with normal blood pressure can effectively reduce their blood pressure through exercise [49,84,85], and this effect is independent of weight loss [86]. This is also consistent with our analysis results. Compared with pre-intervention, HT significantly improved SBP (MD = −5.19, *p* = 0.00) and DBP (MD = −3.25, *p* = 0.00) in overweight or obese adults. Additionally, in our study, the baseline blood pressure values of these overweight or obese adults were all within the normal range. However, in the comparative analysis with NT, no significant advantage of HT was observed, which aligns with the findings of previous studies [23]. 

HT does not provide additional benefits in terms of improving blood pressure. The development of hypertension is closely related to excessive activity of the sympathetic nervous system [87]. For individuals with hypertension, the increased production of nitric oxide and enhanced bioavailability triggered by exercise may serve as the primary physiological mechanisms for lowering blood pressure [88,89]. Obesity exacerbates the risk of cardiometabolic diseases and is also a significant risk factor for hypertension [90]. Currently, in addition to long-term reliance on medication to control blood pressure, exercise has been recommended as an effective approach to prevent elevated blood pressure [91]. Research has shown that HIIT can significantly improve blood pressure in overweight or obese individuals by approximately 3–5 mmHg [91]. HIIT had the most beneficial effect on improving CRF in overweight and obese adults [74], which corresponds to one aspect of our recommendation: encouraging overweight or obese adults to engage in HIIT to improve cardiometabolic health. The potential physiological mechanisms by which high-intensity exercise improves blood pressure may be related to the observed enhancements in vascular endothelial function and autonomic nervous system regulation during high-intensity exercise interventions [30]. Research has indicated that, compared with other types of exercise, combined and hybrid training can yield the most beneficial effects on cardiometabolic health indicators [30]. Although our study confirmed that HT can improve blood pressure in overweight or obese adults, the effects of hypoxic and normoxic environments on blood pressure improvement were similar. The impact of combining hypoxic stimulation with exercise on blood pressure remains unclear. Future research should focus on exploring the potential of combining different types of exercise with hypoxic training paradigms to determine the possible effects of HT on improving cardiometabolic health indicators. This will help continuously optimize the best exercise prescriptions for HT to enhance cardiometabolic health in overweight or obese adults.

Limitations: Among the 17 included studies, 6 reported varying degrees of participant attrition, and 2 studies had sample sizes of fewer than 10 participants. The types of exercise examined in the included studies were diverse; however, after further categorization, certain exercise types, such as combined training and Pilates, were represented by only one study each. This limitation hindered our ability to perform more detailed subgroup analyses across different exercise types. Among the included studies, the subjects in eight studies were analyzed as a combined group of males and females. Therefore, it was not possible to conduct a subgroup analysis by gender. In terms of age, in this study, 45 years old was taken as the benchmark, and the subjects were divided into young individuals and middle-aged and elderly individuals. Due to the limitation of the number of studies, there was no further distinction made between middle-aged and elderly individuals. The prescription of exercise intensity has always been a challenging task [92]. Owing to the limited number of studies, we were unable to explore the exercise intensity prescription for HIIT under hypoxic conditions in overweight or obese adults; instead, we only suggested that the intervention duration should be less than 45 min. Future research should prioritize determining the optimal exercise intensity prescription for HIIT under hypoxic conditions in overweight or obese adults.

## 5. Conclusions

HT demonstrates favorable effects in improving cardiometabolic health among overweight or obese adults. Compared with NT, this advantage is primarily reflected in CRF, while the effects on SBP and DBP are similar. It is recommended that young adults who are overweight or obese engage in HIIT under hypoxic conditions (FiO_2_ > 15%). The recommended intervention should span 8 weeks, with three sessions per week, and each session should not exceed 45 min.

## Data Availability

All data generated or analyzed during this study are included in the article/Supplementary Materials.

## Supplementary Materials

The following supporting information can be downloaded at https://www.mdpi.com/article/10.3390/life15040566/s1: Table S1: Search strategy; Table S2: Main characteristics of studies included in the meta-analysis; Table S3: Certainty of evidence for meta-analyzed outcomes.

## References

[1] Breen C., O’Connell J., Geoghegan J., O’Shea D., Birney S., Tully L., Gaynor K., O’Kelly M., O’Malley G., O’Donovan C. (2022). Obesity in Adults: A 2022 Adapted Clinical Practice Guideline for Ireland. Obes. Facts.

[2] Collaborators G.A.B. (2025). Global, regional, and national prevalence of adult overweight and obesity, 1990-2021, with forecasts to 2050: A forecasting study for the Global Burden of Disease Study 2021. Lancet.

[3] Badimon L., Bugiardini R., Cenko E., Cubedo J., Dorobantu M., Duncker D.J., Estruch R., Milicic D., Tousoulis D., Vasiljevic Z. (2017). Position paper of the European Society of Cardiology-working group of coronary pathophysiology and microcirculation: Obesity and heart disease. Eur. Heart J..

[4] Myers J., McAuley P., Lavie C.J., Despres J.P., Arena R., Kokkinos P. (2015). Physical activity and cardiorespiratory fitness as major markers of cardiovascular risk: Their independent and interwoven importance to health status. Prog. Cardiovasc. Dis..

[5] Ghaith A., Chacaroun S., Borowik A., Chatel L., Doutreleau S., Wuyam B., Tamisier R., Pépin J.L., Flore P., Verges S. (2022). Hypoxic high-intensity interval training in individuals with overweight and obesity. Am. J. Physiol. Regul. Integr. Comp. Physiol..

[6] Caspersen C.J., Powell K.E., Christenson G.M. (1985). Physical activity, exercise, and physical fitness: Definitions and distinctions for health-related research. Public Health Rep..

[7] Harber M.P., Kaminsky L.A., Arena R., Blair S.N., Franklin B.A., Myers J., Ross R. (2017). Impact of Cardiorespiratory Fitness on All-Cause and Disease-Specific Mortality: Advances Since 2009. Prog. Cardiovasc. Dis..

[8] Kodama S., Saito K., Tanaka S., Maki M., Yachi Y., Asumi M., Sugawara A., Totsuka K., Shimano H., Ohashi Y. (2009). Cardiorespiratory fitness as a quantitative predictor of all-cause mortality and cardiovascular events in healthy men and women: A meta-analysis. Jama.

[9] Malhotra R., Bakken K., D’Elia E., Lewis G.D. (2016). Cardiopulmonary Exercise Testing in Heart Failure. JACC. Heart Fail..

[10] Kabbadj K., Taiek N., El Hjouji W., El Karrouti O., El Hangouche A.J. (2024). Cardiopulmonary Exercise Testing: Methodology, Interpretation, and Role in Exercise Prescription for Cardiac Rehabilitation. US Cardiol..

[11] Ekblom-Bak E., Ekblom B., Söderling J., Börjesson M., Blom V., Kallings L.V., Hemmingsson E., Andersson G., Wallin P., Ekblom Ö. (2019). Sex- and age-specific associations between cardiorespiratory fitness, CVD morbidity and all-cause mortality in 266.109 adults. Prev. Med..

[12] Otsuki T., Kotato T., Zempo-Miyaki A. (2016). Habitual exercise decreases systolic blood pressure during low-intensity resistance exercise in healthy middle-aged and older individuals. Am. J. Physiol. Heart Circ. Physiol..

[13] Rahmati M., Lee H., Lee H., Park J., Vithran D.T.A., Li Y., Kazemi A., Boyer L., Fond G., Smith L. (2025). Associations Between Exercise Training, Physical Activity, Sedentary Behaviour and Mortality: An Umbrella Review of Meta-Analyses. J. Cachexia Sarcopenia Muscle.

[14] Atakan M.M., Türkel İ., Özerkliğ B., Koşar Ş.N., Taylor D.F., Yan X., Bishop D.J. (2024). Small peptides: Could they have a big role in metabolism and the response to exercise?. J. Physiol..

[15] Atakan M.M., Koşar Ş.N., Güzel Y., Tin H.T., Yan X. (2021). The Role of Exercise, Diet, and Cytokines in Preventing Obesity and Improving Adipose Tissue. Nutrients.

[16] Weiner R.B., Baggish A.L. (2012). Exercise-induced cardiac remodeling. Prog. Cardiovasc. Dis..

[17] Chen Z., Zhou R., Liu X., Wang J., Wang L., Lv Y., Yu L. (2025). Effects of Aerobic Exercise on Blood Lipids in People with Overweight or Obesity: A Systematic Review and Meta-Analysis of Randomized Controlled Trials. Life.

[18] Callahan M.J., Parr E.B., Hawley J.A., Camera D.M. (2021). Can High-Intensity Interval Training Promote Skeletal Muscle Anabolism?. Sports Med..

[19] Gibala M.J., McGee S.L. (2008). Metabolic adaptations to short-term high-intensity interval training: A little pain for a lot of gain?. Exerc. Sport Sci. Rev..

[20] MacInnis M.J., Gibala M.J. (2017). Physiological adaptations to interval training and the role of exercise intensity. J. Physiol..

[21] Urdampilleta A., González-Muniesa P., Portillo M.P., Martínez J.A. (2012). Usefulness of combining intermittent hypoxia and physical exercise in the treatment of obesity. J. Physiol. Biochem..

[22] Oppert J.M., Bellicha A., van Baak M.A., Battista F., Beaulieu K., Blundell J.E., Carraça E.V., Encantado J., Ermolao A., Pramono A. (2021). Exercise training in the management of overweight and obesity in adults: Synthesis of the evidence and recommendations from the European Association for the Study of Obesity Physical Activity Working Group. Obes. Rev. Off. J. Int. Assoc. Study Obes..

[23] Ramos-Campo D.J., Girard O., Pérez A., Rubio-Arias J. (2019). Additive stress of normobaric hypoxic conditioning to improve body mass loss and cardiometabolic markers in individuals with overweight or obesity: A systematic review and meta-analysis. Physiol. Behav..

[24] Tee C.C.L., Cooke M.B., Chong M.C., Yeo W.K., Camera D.M. (2023). Mechanisms for Combined Hypoxic Conditioning and Divergent Exercise Modes to Regulate Inflammation, Body Composition, Appetite, and Blood Glucose Homeostasis in Overweight and Obese Adults: A Narrative Review. Sports Med..

[25] Yin M., Li H., Bai M., Liu H., Chen Z., Deng J., Deng S., Meng C., Vollaard N.B.J., Little J.P. (2024). Is low-volume high-intensity interval training a time-efficient strategy to improve cardiometabolic health and body composition? A meta-analysis. Appl. Physiol. Nutr. Metab. Physiol. Appl. Nutr. Metab..

[26] Scharhag-Rosenberger F., Meyer T., Gässler N., Faude O., Kindermann W. (2010). Exercise at given percentages of VO_2_max: Heterogeneous metabolic responses between individuals. J. Sci. Med. Sport.

[27] Kong Z., Zang Y., Hu Y. (2014). Normobaric hypoxia training causes more weight loss than normoxia training after a 4-week residential camp for obese young adults. Sleep Breath. Schlaf Atm..

[28] Camacho-Cardenosa A., Camacho-Cardenosa M., Burtscher M., Martínez-Guardado I., Timon R., Brazo-Sayavera J., Olcina G. (2018). High-Intensity Interval Training in Normobaric Hypoxia Leads to Greater Body Fat Loss in Overweight/Obese Women than High-Intensity Interval Training in Normoxia. Front. Physiol..

[29] Yin M., Xu K., Deng J., Deng S., Chen Z., Zhang B., Zhong Y., Li H., Zhang X., Toledo M.J.L. (2024). Optimal Frequency of Interrupting Prolonged Sitting for Cardiometabolic Health: A Systematic Review and Meta-Analysis of Randomized Crossover Trials. Scand. J. Med. Sci. Sports.

[30] Batrakoulis A., Jamurtas A.Z., Metsios G.S., Perivoliotis K., Liguori G., Feito Y., Riebe D., Thompson W.R., Angelopoulos T.J., Krustrup P. (2022). Comparative Efficacy of 5 Exercise Types on Cardiometabolic Health in Overweight and Obese Adults: A Systematic Review and Network Meta-Analysis of 81 Randomized Controlled Trials. Circulation. Cardiovasc. Qual. Outcomes.

[31] Ashton R.E., Tew G.A., Aning J.J., Gilbert S.E., Lewis L., Saxton J.M. (2020). Effects of short-term, medium-term and long-term resistance exercise training on cardiometabolic health outcomes in adults: Systematic review with meta-analysis. Br. J. Sports Med..

[32] Heinonen I.H., Boushel R., Kalliokoski K.K. (2016). The Circulatory and Metabolic Responses to Hypoxia in Humans—With Special Reference to Adipose Tissue Physiology and Obesity. Front. Endocrinol..

[33] Girard O., Duan R., Suzuki K., Yan X. (2022). Editorial: Hypoxia and exercise: Tissue specific and systemic adaptive responses. Front. Physiol..

[34] Lippl F.J., Neubauer S., Schipfer S., Lichter N., Tufman A., Otto B., Fischer R. (2010). Hypobaric hypoxia causes body weight reduction in obese subjects. Obesity.

[35] Liu P., Chen H., Jiang X., Diaz-Cidoncha Garcia J. (2025). Impact of exercise training in a hypobaric/normobaric hypoxic environment on body composition and glycolipid metabolism in individuals with overweight or obesity: A systematic review and meta-analysis. Front. Physiol..

[36] Gangwar A., Paul S., Ahmad Y., Bhargava K. (2020). Intermittent hypoxia modulates redox homeostasis, lipid metabolism associated inflammatory processes and redox post-translational modifications: Benefits at high altitude. Sci. Rep..

[37] Girard O., Malatesta D., Millet G.P. (2017). Walking in Hypoxia: An Efficient Treatment to Lessen Mechanical Constraints and Improve Health in Obese Individuals?. Front. Physiol..

[38] Roels B., Thomas C., Bentley D.J., Mercier J., Hayot M., Millet G. (2007). Effects of intermittent hypoxic training on amino and fatty acid oxidative combustion in human permeabilized muscle fibers. J. Appl. Physiol..

[39] Lefferts W.K., Babcock M.C., Tiss M.J., Ives S.J., White C.N., Brutsaert T.D., Heffernan K.S. (2016). Effect of hypoxia on cerebrovascular and cognitive function during moderate intensity exercise. Physiol. Behav..

[40] Schega L., Peter B., Brigadski T., Leßmann V., Isermann B., Hamacher D., Törpel A. (2016). Effect of intermittent normobaric hypoxia on aerobic capacity and cognitive function in older people. J. Sci. Med. Sport.

[41] Brocherie F., Girard O., Faiss R., Millet G.P. (2017). Effects of Repeated-Sprint Training in Hypoxia on Sea-Level Performance: A Meta-Analysis. Sports Med..

[42] Robertson E.Y., Saunders P.U., Pyne D.B., Gore C.J., Anson J.M. (2010). Effectiveness of intermittent training in hypoxia combined with live high/train low. Eur. J. Appl. Physiol..

[43] Camacho-Cardenosa A., Camacho-Cardenosa M., Brazo-Sayavera J., Timón R., González-Custodio A., Olcina G. (2020). Repeated sprint in hypoxia as a time-metabolic efficient strategy to improve physical fitness of obese women. Eur. J. Appl. Physiol..

[44] Page M.J., McKenzie J.E., Bossuyt P.M., Boutron I., Hoffmann T.C., Mulrow C.D., Shamseer L., Tetzlaff J.M., Akl E.A., Brennan S.E. (2021). The PRISMA 2020 statement: An updated guideline for reporting systematic reviews. BMJ (Clin. Res. Ed.).

[45] Schünemann H.J., Higgins J.P.T., Vist G.E., Glasziou P.P., Akl E.A., Skoetz N., Guyatt G.H. (2019). Completing ‘Summary of findings’ tables and grading the certainty of the evidence. Cochrane Handbook for Systematic Reviews of Interventions.

[46] Wiesner S., Haufe S., Engeli S., Mutschler H., Haas U., Luft F.C., Jordan J. (2010). Influences of normobaric hypoxia training on physical fitness and metabolic risk markers in overweight to obese subjects. Obesity.

[47] Morishima T., Kurihara T., Hamaoka T., Goto K. (2014). Whole body, regional fat accumulation, and appetite-related hormonal response after hypoxic training. Clin. Physiol. Funct. Imaging.

[48] Kong Z., Shi Q., Nie J., Tong T.K., Song L., Yi L., Hu Y. (2017). High-Intensity Interval Training in Normobaric Hypoxia Improves Cardiorespiratory Fitness in Overweight Chinese Young Women. Front. Physiol..

[49] Klug L., Mähler A., Rakova N., Mai K., Schulz-Menger J., Rahn G., Busjahn A., Jordan J., Boschmann M., Luft F.C. (2018). Normobaric hypoxic conditioning in men with metabolic syndrome. Physiol. Rep..

[50] Jung K., Kim J., Park H.Y., Jung W.S., Lim K. (2020). Hypoxic Pilates Intervention for Obesity: A Randomized Controlled Trial. Int. J. Environ. Res. Public Health.

[51] Jiao X., Liu M., Li R., Li J., Wang L., Niu G., Wang L., Ji X., Lv C., Guo X. (2024). Helpful to Live Healthier? Intermittent Hypoxic/Ischemic Training Benefits Vascular Homeostasis and Lipid Metabolism with Activating SIRT1 Pathways in Overweight/Obese Individuals. Obes. Facts.

[52] Hein M., Chobanyan-Jürgens K., Tegtbur U., Engeli S., Jordan J., Haufe S. (2021). Effect of normobaric hypoxic exercise on blood pressure in old individuals. Eur. J. Appl. Physiol..

[53] Gutwenger I., Hofer G., Gutwenger A.K., Sandri M., Wiedermann C.J. (2015). Pilot study on the effects of a 2-week hiking vacation at moderate versus low altitude on plasma parameters of carbohydrate and lipid metabolism in patients with metabolic syndrome. BMC Res. Notes.

[54] Gatterer H., Haacke S., Burtscher M., Faulhaber M., Melmer A., Ebenbichler C., Strohl K.P., Högel J., Netzer N.C. (2015). Normobaric Intermittent Hypoxia over 8 Months Does Not Reduce Body Weight and Metabolic Risk Factors--a Randomized, Single Blind, Placebo-Controlled Study in Normobaric Hypoxia and Normobaric Sham Hypoxia. Obes. Facts.

[55] Chacaroun S., Borowik A., Vega-Escamilla Y.G.I., Doutreleau S., Wuyam B., Belaidi E., Tamisier R., Pepin J.L., Flore P., Verges S. (2020). Hypoxic Exercise Training to Improve Exercise Capacity in Obese Individuals. Med. Sci. Sports Exerc..

[56] Park W., Park H.Y., Kim S.W. (2024). Effects of 12 Weeks of Combined Exercise Training in Normobaric Hypoxia on Arterial Stiffness, Inflammatory Biomarkers, and Red Blood Cell Hemorheological Function in Obese Older Women. Healthcare.

[57] Park H.-Y., Lim K. (2017). The Effects of Aerobic Exercise at Hypoxic Condition during 6 Weeks on Body Composition, Blood Pressure, Arterial Stiffness, and Blood Lipid Level in Obese Women. Int. J. Sports Sci..

[58] Hobbins L., Hunter S., Gaoua N., Girard O. (2021). Short-Term Perceptually Regulated Interval-Walk Training in Hypoxia and Normoxia in Overweight-to-Obese Adults. J. Sports Sci. Med..

[59] Camacho-Cardenosa A., Camacho-Cardenosa M., Brazo-Sayavera J., Burtscher M., Timón R., Olcina G. (2018). Effects of High-Intensity Interval Training Under Normobaric Hypoxia on Cardiometabolic Risk Markers in Overweight/Obese Women. High Alt. Med. Biol..

[60] Yu Q., Kong Z., Zou L., Chapman R., Shi Q., Nie J. (2023). Comparative efficacy of various hypoxic training paradigms on maximal oxygen consumption: A systematic review and network meta-analysis. J. Exerc. Sci. Fit..

[61] Deng L., Liu Y., Chen B., Hou J., Liu A., Yuan X. (2025). Impact of Altitude Training on Athletes’ Aerobic Capacity: A Systematic Review and Meta-Analysis. Life.

[62] Westmacott A., Sanal-Hayes N.E.M., McLaughlin M., Mair J.L., Hayes L.D. (2022). High-Intensity Interval Training (HIIT) in Hypoxia Improves Maximal Aerobic Capacity More Than HIIT in Normoxia: A Systematic Review, Meta-Analysis, and Meta-Regression. Int. J. Environ. Res. Public Health.

[63] Xia X., Hu Y., Wang H., Zheng H., Zhang Y. (2012). A Meta-Analysis on Influence of Intermittent Hypoxia Training on Athlete’s Aerobic Endurance. J. Shanghai Univ. Sport.

[64] Pan X. (2008). Effects of Altitude Training on Middle-and-Long Distance Runners’ Oxygen-Carrying Ability of Blood. J. Beijing Sport Univ..

[65] Dufour S.P., Ponsot E., Zoll J., Doutreleau S., Lonsdorfer-Wolf E., Geny B., Lampert E., Flück M., Hoppeler H., Billat V. (2006). Exercise training in normobaric hypoxia in endurance runners. I. Improvement in aerobic performance capacity. J. Appl. Physiol..

[66] Brazo-Sayavera J., Camacho-Cardenosa A., Morais Fernandes T., Argolo J.G.M., Morais Fernandes A.P., Sorgi C.A., Lizzi E.A.d.S., Trapé Á.A. (2025). Effects of Moderate-Intensity Cyclic Normobaric Hypoxic Training on Cardiovascular Disease Risk Factors of Patients Recovered from COVID-19: The AEROBICOVID Randomized Controlled Trial. High Alt. Med. Biol..

[67] Ruggiero L., Harrison S.W.D., Rice C.L., McNeil C.J. (2022). Neuromuscular fatigability at high altitude: Lowlanders with acute and chronic exposure, and native highlanders. Acta Physiol..

[68] Hasegawa J.S., Silveira A.C., Azevedo R.A., Schamne J.C., Rondon M., Papoti M., Lima-Silva A.E., Koehle M.S., Bertuzzi R. (2025). No sex differences in performance and perceived fatigability during a self-paced endurance exercise performed under moderate hypoxia. Am. J. Physiol. Regul. Integr. Comp. Physiol..

[69] Haufe S., Wiesner S., Engeli S., Luft F.C., Jordan J. (2008). Influences of normobaric hypoxia training on metabolic risk markers in human subjects. Med. Sci. Sports Exerc..

[70] González-Muniesa P., Lopez-Pascual A., de Andrés J., Lasa A., Portillo M.P., Arós F., Durán J., Egea C.J., Martinez J.A. (2015). Impact of intermittent hypoxia and exercise on blood pressure and metabolic features from obese subjects suffering sleep apnea-hypopnea syndrome. J. Physiol. Biochem..

[71] Wenger R.H. (2002). Cellular adaptation to hypoxia: O_2_-sensing protein hydroxylases, hypoxia-inducible transcription factors, and O_2_-regulated gene expression. FASEB J. Off. Publ. Fed. Am. Soc. Exp. Biol..

[72] Scudder M.R., Lambourne K., Drollette E.S., Herrmann S.D., Washburn R.A., Donnelly J.E., Hillman C.H. (2014). Aerobic capacity and cognitive control in elementary school-age children. Med. Sci. Sports Exerc..

[73] Camacho-Cardenosa A., Camacho-Cardenosa M., Olcina G., Timón R., Brazo-Sayavera J. (2019). Detraining effect on overweight/obese women after high-intensity interval training in hypoxia. Scand. J. Med. Sci. Sports.

[74] Wang H., Cheng R., Xie L., Hu F. (2023). Comparative efficacy of exercise training modes on systemic metabolic health in adults with overweight and obesity: A network meta-analysis of randomized controlled trials. Front. Endocrinol..

[75] Li J., Li Y., Atakan M.M., Kuang J., Hu Y., Bishop D.J., Yan X. (2020). The Molecular Adaptive Responses of Skeletal Muscle to High-Intensity Exercise/Training and Hypoxia. Antioxidants.

[76] Williams C.J., Gurd B.J., Bonafiglia J.T., Voisin S., Li Z., Harvey N., Croci I., Taylor J.L., Gajanand T., Ramos J.S. (2019). A Multi-Center Comparison of O(2peak) Trainability Between Interval Training and Moderate Intensity Continuous Training. Front. Physiol..

[77] Gibala M.J., Little J.P., Macdonald M.J., Hawley J.A. (2012). Physiological adaptations to low-volume, high-intensity interval training in health and disease. J. Physiol..

[78] Sabag A., Little J.P., Johnson N.A. (2022). Low-volume high-intensity interval training for cardiometabolic health. J. Physiol..

[79] Zhang H., Tong T.K., Qiu W., Zhang X., Zhou S., Liu Y., He Y. (2017). Comparable Effects of High-Intensity Interval Training and Prolonged Continuous Exercise Training on Abdominal Visceral Fat Reduction in Obese Young Women. J. Diabetes Res..

[80] Wewege M., van den Berg R., Ward R.E., Keech A. (2017). The effects of high-intensity interval training vs. moderate-intensity continuous training on body composition in overweight and obese adults: A systematic review and meta-analysis. Obes. Rev. Off. J. Int. Assoc. Study Obes..

[81] Andreato L.V., Esteves J.V., Coimbra D.R., Moraes A.J.P., de Carvalho T. (2019). The influence of high-intensity interval training on anthropometric variables of adults with overweight or obesity: A systematic review and network meta-analysis. Obes. Rev. Off. J. Int. Assoc. Study Obes..

[82] Wang K., Zhu Y., Wong S.H., Chen Y., Siu P.M., Baker J.S., Sun F. (2021). Effects and dose-response relationship of high-intensity interval training on cardiorespiratory fitness in overweight and obese adults: A systematic review and meta-analysis. J. Sports Sci..

[83] Rugbeer N., Constantinou D., Torres G. (2021). Comparison of High-Intensity Training Versus Moderate-Intensity Continuous Training on Cardiorespiratory Fitness and Body Fat Percentage in Persons With Overweight or Obesity: A Systematic Review and Meta-Analysis of Randomized Controlled Trials. J. Phys. Act. Health.

[84] Al-Mhanna S.B., Batrakoulis A., Wan Ghazali W.S., Mohamed M., Aldayel A., Alhussain M.H., Afolabi H.A., Wada Y., Gülü M., Elkholi S. (2024). Effects of combined aerobic and resistance training on glycemic control, blood pressure, inflammation, cardiorespiratory fitness and quality of life in patients with type 2 diabetes and overweight/obesity: A systematic review and meta-analysis. PeerJ.

[85] Saco-Ledo G., Valenzuela P.L., Ruiz-Hurtado G., Ruilope L.M., Lucia A. (2020). Exercise Reduces Ambulatory Blood Pressure in Patients With Hypertension: A Systematic Review and Meta-Analysis of Randomized Controlled Trials. J. Am. Heart Assoc..

[86] Arroll B., Beaglehole R. (1992). Does physical activity lower blood pressure: A critical review of the clinical trials. J. Clin. Epidemiol..

[87] Grassi G., Mark A., Esler M. (2015). The sympathetic nervous system alterations in human hypertension. Circ. Res..

[88] Pal S., Radavelli-Bagatini S., Ho S. (2013). Potential benefits of exercise on blood pressure and vascular function. J. Am. Soc. Hypertens. JASH.

[89] Whelton S.P., Chin A., Xin X., He J. (2002). Effect of aerobic exercise on blood pressure: A meta-analysis of randomized, controlled trials. Ann. Intern. Med..

[90] Lyall D.M., Celis-Morales C., Ward J., Iliodromiti S., Anderson J.J., Gill J.M.R., Smith D.J., Ntuk U.E., Mackay D.F., Holmes M.V. (2017). Association of Body Mass Index With Cardiometabolic Disease in the UK Biobank: A Mendelian Randomization Study. JAMA Cardiol..

[91] Clark T., Morey R., Jones M.D., Marcos L., Ristov M., Ram A., Hakansson S., Franklin A., McCarthy C., De Carli L. (2020). High-intensity interval training for reducing blood pressure: A randomized trial vs. moderate-intensity continuous training in males with overweight or obesity. Hypertens. Res. Off. J. Jpn. Soc. Hypertens..

[92] Li Y., Li J., Atakan M.M., Wang Z., Hu Y., Nazif M., Zarekookandeh N., Ye H.Z., Kuang J., Ferri A. (2022). Methods to match high-intensity interval exercise intensity in hypoxia and normoxia—A pilot study. J. Exerc. Sci. Fit..

