# Supplementary material for: The Impact of Exercise Training in a Hypobaric/Normobaric Hypoxic Environment on Cardiometabolic Health in Adults with Overweight or Obesity: A Systematic Review and Meta-Analysis
Source: Life (Basel). 2025 Mar 31;15(4):566. doi: 10.3390/life15040566 (PMC12028800; doi:10.3390/life15040566)
Supplement: Supplementary file 1 [file life-15-00566-s001.zip › Supplemental material.pdf]

Supplemental material

Impact of exercise training in a hypobaric/normobaric hypoxic environment on cardiometabolic health in adults with overweight or obesity: a systematic review and meta-analysis

Table S1. Search strategy 2

Table S2. Main characteristics of studies included in the meta-analysis 3

Table S3. Certainty of evidence for meta-analysed outcomes 5

Table S1. Search strategy

Databases	Search strategy	Results
Pub Med	(((((((Intermittent hypoxia) OR (hypoxia exercise)) OR (hypoxic training)) OR (altitude training)) OR (normobaric hypoxia training)) OR (Live high-train high)) OR (Live high-train low)) OR (Live low-train high)) AND (((overweight) OR (obese)) OR (obesity))	859
Web of science	((((((((TS=(Intermittent hypoxia)) OR TS=(hypoxia exercise)) OR TS=(hypoxic training)) OR TS=(altitude training)) OR TS=(normobaric hypoxia training)) OR TS=(Live high-train high)) OR TS=(Live high-train low)) OR TS=(Live low-train high))<br><br>(((TS=(overweight)) OR TS=(obese)) OR TS=(obesity))<br><br>#1 AND #2	1451
EMBASE	'intermittent hypoxia'/exp OR 'intermittent hypoxia' OR (intermittent AND ('hypoxia'/exp OR hypoxia)) OR 'hypoxia exercise' OR (('hypoxia'/exp OR hypoxia) AND ('exercise'/exp OR exercise)) OR 'hypoxic training'/exp OR 'hypoxic training' OR (hypoxic AND ('training'/exp OR training)) OR 'altitude training' OR (('altitude'/exp OR altitude) AND ('training'/exp OR training)) OR 'normobaric hypoxia training' OR (normobaric AND ('hypoxia'/exp OR hypoxia) AND ('training'/exp OR training)) OR 'live high-train high' OR (live AND 'high train' AND high) OR 'live high-train low' OR (live AND 'high train' AND low) OR 'live low-train high' OR (live AND 'low train' AND high)<br>'overweight'/exp OR overweight OR obese OR 'obesity'/exp OR obesity<br>#1 AND #2	1678
Scopus	((TITLE-ABS-KEY ((intermittent AND hypoxia)) OR TITLE-ABS-KEY (hypoxia AND exercise) OR TITLE-ABS-KEY (hypoxic AND training) OR TITLE-ABS-KEY (altitude AND training) OR TITLE-ABS-KEY (normobaric AND hypoxia AND training) OR TITLE-ABS-KEY (live AND high-train AND high) OR TITLE-ABS-KEY (live AND high-train AND low) OR TITLE-ABS-KEY (live AND low-train AND high))) AND ((TITLE-ABS-KEY (overweight) OR TITLE-ABS-KEY (obese) OR TITLE-ABS-KEY (obesity)))	1177
Cochrane Library	((Intermittent hypoxia) OR (hypoxia exercise) OR (hypoxic training) OR (altitude training) OR (normobaric hypoxia training)) AND ((obese) OR (obesity) OR (overweight))	144

Table S2. Main characteristics of studies included in the meta-analysis

Study	Basic information			Intervention program				Outcome
	Participants (M/F)	Age (M±SD)/year	BMI (M±SD)/kg/m^2	Duration/ Frequency	Altitude/ FiO_2	Modality/ Exercise intensity	time	
1(Wiesner et al., 2010)	HT: 10/14<br>NT: 8/13	42.2 ± 1.2<br>42.1 ± 1.7	33.1 ± 0.3<br>32.5 ± 0.8	4 weeks<br>3days/wk	15%	aerobic exercise.<br>65% VO_{2max}	60min	CRF, SBP, DBP
2(Morishima et al., 2014)	HT: 9/0<br>NT: 11/0	30 ± 2<br>32 ± 3	25.6 ± 1.2<br>25.4 ± 0.9	4 weeks<br>3days/wk	15%	aerobic exercise.<br>55% VO_{2max}	60min	CRF
3(Kong et al., 2014)	HT: 5/5<br>NT: 5/3	19.8 ± 2.2<br>22.3 ± 1.7	34.7 ± 5.3<br>33.8 ± 5.6	4 weeks<br>7days/wk	16.4 - 14.5%	Aerobic and resistance training.<br>60%-70%HRmax	60min	SBP, DBP
4(Gatterer et al., 2015)	HT: 4/12<br>NT: 10/6	50.3 ± 10.3<br>52.4 ± 7.9	37.9 ± 8.1<br>36.3 ± 4.2	a: 3 months<br>b: 8 months<br>2 days/wk	3500m	aerobic exercise.<br>65-70% HRmax	90min	CRF, SBP, DBP
5(Gutwenger et al., 2015)	HT: 3/5<br>NT: 3/3	50.1 ± 7.8<br>63.3 ± 5.2	31.1 ± 5.3<br>32.3 ± 4.2	2weeks<br>4days/wk	1900m	hiking<br>55%-65%HRmax	180min	CRF, SBP, DBP
6(Kong et al., 2017)	HT: 0/11<br>NT: 0/13	18 - 30	26.0 ± 2.4<br>25.7 ± 2.2	5weeks<br>4days/wk	15%	60 repetitions of 8 s maximal cycling effort interspersed with 12-s	<45min	CRF
7(Park and Lim, 2017)	HTa: 0/11<br>HTb: 0/12<br>NT: 0/12	42.0 ± 4.4<br>46.6 ± 5.7<br>47.2 ± 6.3	>30	6 weeks<br>5 days/wk	a: 16.5%<br>b: 14.5%	aerobic exercise.<br>75% HRmax	60min	SBP, DBP
8(Camacho-Cardenosa et al., 2018a)	HT: 0/13<br>NT: 0/15	44.43 ± 7.18<br>43.14 ± 7.67	30.03 ± 6.37<br>29.59 ± 5.25	a1: 6weeks<br>a2: 12weeks<br>3 days/wk	17.2%	High intensity interval training	<45min	SBP, DBP
8(Camacho-Cardenosa et al., 2018a)	HT: 0/15<br>NT: 0/18	37.4 ± 10.25<br>40.05 ± 8.66	27.71 ± 4.55<br>28.74 ± 4.77	b1: 6weeks<br>b2: 12weeks<br>3 days/wk	17.2%	High intensity full sprint	<45min	
9(Klug et al., 2018)	HT: 12/0<br>NT: 11/0	55.0 ± 2.1<br>57.6 ± 2.2	35.5 ± 1.4<br>34.1 ± 0.9	6 weeks<br>3 days/wk	15%	aerobic exercise.<br>50-60% HRmax	60min	CRF, SBP, DBP

10(Camacho-Cardenosa et al., 2020)	HT: 0/19<br>NT: 0/20	unspecified	29.78 ± 6.23<br>29.31 ± 5.21	12weeks<br>3 days/wk	17.2%	High intensity interval training	<45min	CRF
10(Camacho-Cardenosa et al., 2020)	HT: 0/22<br>NT: 0/21	unspecified	27.71 ± 4.55<br>28.28 ± 4.47	12weeks<br>3 days/wk	17.2%	High intensity full sprint	<45min	
11(Chacaroun et al., 2020)	HT: 11/1<br>NT: 8/3	52 ± 12<br>56 ± 11	31.2 ± 2.4<br>31.8 ± 3.2	8 weeks<br>3 days/wk	13%	aerobic exercise.<br>75% VO_{2max}	45min	CRF, SBP, DBP
12(Jung et al., 2020)	HT: 0/12<br>NT: 0/10	47.2 ± 6.4<br>43.8 ± 8.6	27.1 ± 4.3<br>25.1 ± 3.3	12 weeks<br>3 days/wk	14.5%	Pilates training.	50min	CRF, SBP, DBP
13(Hein et al., 2021)	HT: 12<br>NT: 13	60.4 ± 5.0<br>63.8 ± 5.8	28.6 ± 3.0<br>28.4 ± 1.9	8weeks<br>3 days/wk	15%	aerobic exercise.<br>VO_{2max}60%-70%	30-40min	CRF, SBP, DBP
14(Hobbins et al., 2021)	HT: 4/4<br>NT: 5/3	32.1 ± 10.2<br>41.1 ± 13.0	31.9 ± 3.6<br>33.0 ± 1.4	2 weeks<br>4 days/wk	13%	self-paced interval-walking training.<br>RPE: 14	60min	SBP, DBP
15(Ghaith et al., 2022)	HT: 10/6<br>NT: 13/2	51.0 ± 8.3<br>52.0 ± 7.5	31.5 ± 4.0<br>32.4 ± 4.8	8 weeks<br>3 days/wk	12%	cycling at 80% or 100% of maximal workload	60min	CRF, SBP, DBP
16(Jiao et al., 2024)	HT: 5/8<br>NT: 5/4	36.62 ± 9.54<br>31.33 ± 8.75	28.08 ± 2.10<br>29.28 ± 5.81	4weks<br>5 days/wk	15%	aerobic exercise.<br>70% VO_{2max}	60min	CRF, SBP, DBP
17(Park et al., 2024)	HT: 0/12<br>NT: 0/12	67.83 ± 1.03<br>68.08 ± 0.90	27.31 ± 0.66<br>26.87 ± 0.38	12 weeks<br>3 days/wk	14.5%	resistance and aerobic training.<br>60-70% HRmax	60min	SBP, DBP

Note: M, male; F, female; wk, weeks; HRmax, max heart rate; VO2max, maximal oxygen consumption; CRF, cardiorespiratory fitness, including VO_{2max}/VO_{2peak}; SBP, Systolic Blood Pressure; DBP, Diastolic Blood Pressure.

Table S3. Certainty of evidence for meta-analysed outcomes

Outcome	No of Participants and Studies	Gradeassessment					Certainty of evidence
		Risk of bias	Inconsistency	Indirectness	Imprecision	Publication bias	
CRF (H - post, H - pre)	404 (14 RCTs)	Serious[a]	Not serious	Not serious	Serious[c]	Not serious	Low
CRF (H - post, N - post)	391 (14 RCTs)	Serious[a]	Not serious	Not serious	Serious[d]	Not serious	Low
SBP (H - post, H - pre)	486 (19 RCTs)	Serious[a]	Not serious	Not serious	Serious[c]	Not serious	Low
SBP (H - post, N - post)	487 (19 RCTs)	Serious[a]	Not serious	Not serious	Serious[c]	Not serious	Low
DBP (H - post, H - pre)	486 (19 RCTs)	Serious[a]	Not serious	Not serious	Not serious	Not serious	Moderate
DBP (H - post, N - post)	487 (19 RCTs)	Serious[a]	Serious[b]	Not serious	Serious[c]	Not serious	Very low

a: High risk of bias with allocation concealment.

b: High heterogeneity.

c: The confidence intervals indicated the potential for import harm or benefit.

d: Sample size not more than 400.
